# Draft Genome Sequence of a *Legionella pneumophila* Serogroup 4 Strain Causing Legionellosis

**DOI:** 10.1128/genomeA.00602-14

**Published:** 2014-06-19

**Authors:** Akira Okamoto, Hiromichi Lee, Mitsutaka Yabutani, Keiko Yamada, Michio Ohta

**Affiliations:** a Department of School Health Sciences, Aichi University of Education, Kariya, Japan; b Department of Bacteriology, Nagoya University Graduate School of Medicine, Showa-ku, Nagoya, Japan; c Nagoya City Public Health Research Institute, Mizuho-ku, Nagoya, Japan; d School of Nursing, Sugiyama Jogakuen University, Chikusa-ku, Nagoya, Japan

## Abstract

Here, we report the draft genome sequence of the *Legionella pneumophila* Nagoya-1 strain, serogroup 4, which was isolated from a clinical sample from a patient with legionellosis. Several virulence-associated genes, including those encoding the type IV (Dot/Icm) secretion system and effector proteins, were highly conserved.

## GENOME ANNOUNCEMENT

*Legionella pneumophila* is the causative agent of legionellosis, including pneumonia and Pontiac fever, and it is transferred by waterborne aerosolized bacteria rather than human-to-human interaction. *L. pneumophila* strains are categorized by serogroups, on the basis of the lipopolysaccharide cell surface structure. Serogroup 1 has been well investigated, because it is the predominant serogroup among clinical isolates (1, 2). Although other serogroups are often isolated, few have been investigated (3, 4).

Here, we report a draft genome sequence of *L. pneumophila* serogroup 4 strain Nagoya-1. This strain was isolated in 2000 from a patient with legionellosis in a hospital in Japan. Simultaneously, another isolate of *L. pneumophila* serogroup 4 was obtained from a bathroom in the hospital. The two isolates yielded comparable pulsed-field gel electrophoresis patterns.

Genomic DNA was extracted from an overnight culture of *L. pneumophila* Nagoya-1 by using a Wizard genomic DNA purification kit (Promega, Madison, WI). A DNA library was prepared for sequencing with a DNA sample prep kit (Illumina, San Diego, CA), according to the supplied protocol. Single-end sequencing with a read length of 36 bp was performed using a genome analyzer system (Illumina). We obtained 6,130,067 reads that passed the quality test, and the total amount of read data comprises 220,682,412 bp, representing approximately 60-fold coverage of the genome. The read sequences were compiled using Edena version 2.1.1 software (5). The assembled genome consists of 426 contigs, with a total length of 3,363,464 bp and 38.2% average G+C content. The genome sequence was annotated by the Rapid Annotations using Subsystems Technology (RAST) server (6). In all, 3,221 features were identified in 426 contigs, consisting of 3,185 predicted coding sequences, 33 tRNA genes, and 3 rRNAs. tRNA predictions were confirmed by the tRNAscan-SE and RNAmmer servers (7, 8), revealing results consistent with those from the RAST server. The contigs were mapped as a query to the reference genome, *L. pneumophila* strain Philadelphia 1 (serogroup 1, 3,397,754 bp), using the MUMmer (version 3.0) program; however, only 701,725 bp (20.7% of the reference genome) was covered with the contigs.

Genes encoding the type IV (Dot/Icm) secretion system and several effector proteins were annotated. A homology search analysis was performed with the nucleotide sequences of genes encoding the type IV secretion system and effector proteins from the Philadelphia 1 strain as queries, and contigs of Nagoya-1 were used as the database. All of the genes in the Philadelphia 1 *dot/icm* loci were found in the Nagoya-1 genome, with nucleotide sequence identities of 84 to 100%. Genes encoding several effectors, including *legL3*, *legLC8*, *lepA*, *lepB*, *lepB-1*, *shdA*, *sidF*, *vipA*, and *vipF*, were confirmed, with identities of 95.0 to 99.5%.

### Nucleotide sequence accession numbers.

This whole-genome shotgun project has been deposited in the DNA Data Bank of Japan (DDBJ), European Molecular Biology Laboratory, and GenBank under the accession numbers BAZA01000001 to BAZA01000426. The run data generated from sequencing were deposited in the DRA database in the DDBJ under the accession number DRA002158.
